# Green Low-Temperature Activation and Curing for High-Toughness Geopolymer Binders from Diabase Tailings

**DOI:** 10.3390/ma18163815

**Published:** 2025-08-14

**Authors:** Yanan Hu, Yong Yao, Lingling Zhang, Xianming Hu, Xinchun Yang

**Affiliations:** 1 School of Civil Engineering and Architecture, Southwest University of Science and Technology, Mianyang 621010, China; yy001221@163.com; 2 Shock and Vibration of Engineering Materials and Structures Key Laboratory of Sichuan Province, Mianyang 621010, China; 3 Guangyuan Transportation Investment Group Co., Ltd., Guangyuan 628000, China; 4 Sichuan Highway Engineering Consulting and Supervision Co., Ltd., Chengdu 610000, China; 13708073002@163.com

**Keywords:** diabase tailings, geopolymer, low-temperature activation, high toughness, cementitious binder

## Abstract

This study addresses the low reactivity and poor toughness of diabase tailings (DT), a high-silica industrial byproduct, which restricts their large-scale application in geopolymer binders. To overcome these limitations, a dual-regulation strategy integrating stepwise low-temperature thermal activation (100, 200, and 300 °C) with standard curing (20 ± 2 °C, 95% RH) was developed. This approach aimed to enhance mineral dissolution kinetics and facilitate the formation of a dense, interconnected gel network. XRD, FTIR, and SEM analyses revealed significant decomposition of amphibole, pyroxene, and olivine, accompanied by increased release of reactive Si and Al species, leading to the formation of a compact N–A–S–H/C–A–S–H gel structure. Under optimized conditions (Si/Al = 2.6; activator modulus = 1.2), the geopolymer achieved a 7-day compressive strength of 42.3 ± 1.8 MPa, a flexural strength of 12.76 ± 1.6 MPa, and a flexural-to-compressive strength ratio of 0.308, demonstrating significant improvements in toughness compared with conventional binders. This green, energy-efficient strategy not only reduces energy consumption and CO_2_ emissions but also provides a technically feasible pathway for the high-value reuse of silicate-rich mining wastes, contributing to the development of sustainable construction materials with enhanced mechanical performance.

## 1. Introduction

Mine tailings, the residual byproducts of mineral processing, continue to pose serious challenges due to their large volumes, land occupation, ecological risks, and potential release of hazardous substances [1,2]. Among these, diabase tailings (DT)—a silicate-rich industrial byproduct—are produced in quantities exceeding 10 million tons annually in China [3]. Their high SiO_2_ (~49.05 wt%), Al_2_O_3_ (~15.6 wt%), and Fe_2_O_3_ (~16.99 wt%) contents suggest strong potential as precursors for alternative cementitious materials [4]. However, landfilling remains the predominant disposal method, leading to severe underutilization of these resources and aggravating environmental risks. At the same time, Ordinary Portland Cement (OPC), the most widely used construction binder, contributes nearly 8% of global CO_2_ emissions, releasing approximately 900 kg of CO_2_ per ton of cement produced—primarily due to high-temperature calcination [5]. These concerns have driven interest in geopolymers, which offer lower emissions and superior heavy-metal immobilization [6,7,8].

Despite their chemical potential, geopolymers derived from diabase tailings often exhibit inadequate mechanical strength and intrinsic brittleness, largely due to the complex silicate mineralogy of DT [9]. Their reactivity is constrained by the dominance of thermodynamically stable phases such as amphibole, pyroxene, and olivine [10,11]. Conventional activation approaches—including high-temperature calcination (>600 °C) and strong alkali treatments—have been used to disrupt these crystalline frameworks and improve Si/Al dissolution [12,13]. However, these methods are energy-intensive and carbon-heavy. For instance, calcining copper tailings at 600 °C increased reactivity by 54–120%, yet contributed more than 50% of the total system carbon footprint [14]. Similar limitations have been observed in iron, rare-earth, and other high-silica tailings, where low reactivity and efflorescence inhibit the development of robust geopolymer networks [15,16,17]. Furthermore, limited understanding of Fe–O–Si bonding and gel network evolution continues to hinder the optimization of such systems [18,19]. Therefore, cost-effective and energy-efficient strategies are urgently needed to enhance the reactivity of high-silica wastes while maintaining the structural integrity of the resulting binders [20].

Low-temperature thermal activation (100–300 °C) has emerged as a promising approach for improving the reactivity of aluminosilicate-rich materials. This treatment induces interlayer dehydration, hydroxyl elimination, and partial oxidation of Fe^2+^ to Fe^3+^, generating defect-rich amorphous phases and weakening Si–O and Al–O bonds [21,22,23]. These structural changes markedly enhance subsequent alkali reactivity [24,25,26]. For example, a blue shift in the hydroxyl band (from 3359 to 3408 cm^−1^) detected by FTIR after treatment at 300 °C has been directly associated with increased phase reactivity [27]. Additionally, combined low-temperature thermal and alkali pretreatments have been shown to enhance geopolymerization while reducing energy consumption by nearly 50% compared with traditional calcination [28]. The application of standardized test methods, such as GB/T 17671-2021 [26], further ensures the practical compatibility of these materials with existing construction practices [29]. Collectively, these findings provide a strong scientific basis for stepwise low-temperature activation as a viable method for enhancing the performance of complex silicate-rich tailings.

This study proposes a green, low-energy geopolymer binder system developed entirely from diabase tailings. A dual-regulation strategy—combining stepwise low-temperature thermal activation (100, 200, and 300 °C) with standard curing (20 ± 2 °C, 95% RH)—was implemented to enhance mineral dissolution kinetics and facilitate gel network formation. The structural evolution of key crystalline phases during geopolymerization was monitored using XRD, FTIR, and SEM, enabling detailed insights into the dehydroxylation–polycondensation pathway. Moreover, the effects of activation temperature, activator modulus, Si/Al molar ratio, and curing conditions on mechanical performance were systematically evaluated. This approach directly addresses the low reactivity of silicate-rich tailings while reducing energy consumption and CO_2_ emissions, offering a technically feasible pathway for the high-value reuse of diabase tailings in sustainable construction materials.

## 2. Materials and Methods

### 2.1. Raw Materials and Thermal Pretreatment

The diabase tailings (DT) utilized in this study were obtained from Fangshi Town, Qingchuan County, Guangyuan City, Sichuan Province, China. These tailings, a byproduct of the wet beneficiation process of mafic igneous rock, are characterized by high silicate content and limited intrinsic cementitious activity. X-ray fluorescence (XRF) analysis was conducted using a wavelength-dispersive spectrometer (Axios, PANalytical, Almelo, The Netherlands). Measurements were reported on a normalized dry basis excluding loss on ignition (LOI), ensuring that the total oxide content approached 100%. As summarized in Table 1, the DT primarily comprised SiO_2_ (49.05 wt%), Fe_2_O_3_ (16.99 wt%), Al_2_O_3_ (15.60 wt%), CaO (10.05 wt%), and MgO (5.05 wt%), along with minor amounts of TiO_2_, Na_2_O, K_2_O, and trace elements. This composition confirmed the classification of DT as a silico-aluminous solid waste, though it was notably deficient in alkali and reactive alumina components.

X-ray diffraction (XRD) analysis revealed that the major crystalline phases included thermodynamically stable amphiboles—such as magnesiohornblende, tremolite, and pargasite—as well as chlorite-group minerals, including clinochlore and chamosite. Minor quantities of feldspar and quartz were also detected. These mineral phases are generally insoluble in alkaline environments, which collectively contribute to the poor alkali activation potential of raw DT.

To enable direct comparison with ordinary Portland cement, the physical fineness of DT was evaluated using standardized cement testing methods. The residue on a 45 µm square-mesh sieve was determined according to GB/T 1345-2005 [30], utilizing a cement fineness negative-pressure sieving apparatus (HFT-150, Tianjin Hualong Test Instrument Co., Tianjin, China). The Blaine specific surface area was measured using a Blaine air-permeability apparatus (HMP-200, Tianjin Hualong Test Instrument Co., Tianjin, China) in accordance with GB/T 8074-2008 [31], and the bulk density was determined according to GB/T 208-2014 [32]. The DT exhibited a 45 µm sieve residue of 27.76%, significantly higher than that of ordinary Portland cement (≤10%), and a Blaine specific surface area of 6414.66 cm^2^/g, which far exceeds the typical range of 3000–4000 cm^2^/g for cement. The Blaine specific surface area, which reflects the combined effects of particle morphology, distribution, and external surface area [33], was adopted as a key indicator of DT fineness and powder reactivity. The results suggest that DT possesses a bimodal particle size distribution, characterized by the coexistence of ultrafine and coarse particles. This structural feature promotes the dissolution of reactive components while retaining skeletal particles, thereby facilitating the progressive development of the geopolymer matrix.

To enhance the reactivity of DT, a stepwise low-temperature thermal pretreatment was implemented. Samples were oven-dried at 45 °C for at least 4 h to eliminate residual moisture. Thermal activation was then performed at 100 °C, 200 °C, and 300 °C for 3 h using a programmable muffle furnace (SX2 series, Shanghai Yiheng Scientific Instruments Co., Shanghai, China). After heating, samples were rapidly air-cooled at 25 ± 2 °C for 60 min with an airflow rate of 2 m/s to preserve metastable phases and inhibit structural relaxation. The treated powders were subsequently ground using a planetary ball mill (PM100, Retsch GmbH, Haan, Germany) for 1 min and sieved through a 190-mesh screen to obtain uniform particles with an average size of approximately 80 µm. This particle size range was selected based on previous findings [34,35], which indicated that maintaining tailings within the 75–90 µm range offers an effective balance between reactivity, workability, and energy efficiency.

To compensate for the low alumina content and achieve a target Si/Al molar ratio of 2–3, aluminum hydroxide [Al(OH)_3_] was introduced as a supplementary alumina source. Sodium silicate solution (modulus = 2.23) was simultaneously added to enrich the silica content and promote gel network formation. The alkaline activator was formulated by blending sodium hydroxide, sodium silicate, aluminum hydroxide, and deionized water. The composition was adjusted to maintain a SiO_2_/Na_2_O molar ratio of 1.2 and a SiO_2_/Al_2_O_3_ ratio within the range of 2.6–3.0, ensuring optimal conditions for geopolymerization.

DT-based geopolymer slurries were prepared by mixing the thermally activated powder with the prepared alkaline activator at a fixed liquid-to-solid (L/S) ratio of 0.40. The mixture was poured into molds and allowed to set statically for 24 h. After demolding, the specimens were subjected to two curing regimes: ambient curing at 25 ± 2 °C and sealed standard curing at 20 ± 2 °C with 95% relative humidity (RH). All specimens were cured for 7 days prior to further testing and characterization.

### 2.2. Analytical Methods

To systematically assess the influence of thermal activation and formulation parameters on the performance of diabase tailings-based geopolymers (DTG), a four-factor, three-level orthogonal experimental design was adopted. This statistical approach enabled the efficient evaluation of multiple variables while reducing the number of experimental trials required [36]. The four investigated factors included activation temperature, Si/Al molar ratio, activator modulus, and curing condition. The activation temperature variable comprised one unactivated control group (T0) and three thermally activated groups (T1, T2, and T3), corresponding to 100 °C, 200 °C, and 300 °C, respectively. The detailed level combinations and formulation matrix are presented in Table 2.

Statistical evaluation was performed in accordance with GB/T 33568-2017 [37], employing analysis of variance (ANOVA) and range analysis with a confidence level of 95% (α = 0.05). ANOVA was used to quantify the contribution of each factor and to identify statistically significant influences on compressive strength. All data analyses were conducted using SPSS 26.0 (IBM Corp., Armonk, NY, USA), and the corresponding main effect plots and interaction trends were visualized using OriginPro 2024 (OriginLab Corp., Northampton, MA, USA).

Mineralogical transformations induced by thermal activation were characterized using X-ray diffraction (XRD), a well-established technique for identifying crystalline phases and assessing structural disorder in mineral-based geopolymer systems [38]. XRD measurements were performed on an X’Pert PRO diffractometer (PANalytical, Almelo, The Netherlands) equipped with an X’Celerator high-speed detector and a small-angle scattering module. Cu Kα radiation (λ = 1.5406 Å) was applied at 45 kV and 50 mA. Scans were conducted over a 2θ range of 5–80°, with a step size of 0.02° and a scan rate of 2°/min.

Fourier transform infrared spectroscopy (FTIR) was employed to investigate the evolution of functional groups and bonding environments in both the tailings and the geopolymer matrix. Owing to its sensitivity to vibrational modes of hydroxyl, silicate, and aluminate species, FTIR provides valuable insights into thermally induced structural modifications. Spectra were acquired using a Nicolet 5700 spectrometer (Thermo Fisher Scientific, Waltham, MA, USA) fitted with an attenuated total reflectance (ATR) accessory. For each sample, 1 mg of finely ground DT was thoroughly mixed with 100 mg of spectroscopic-grade KBr and pressed into a 13 mm pellet under 10 t of pressure. Each spectrum was recorded over the 4000–400 cm^−1^ range at a resolution of 4 cm^−1^, with 32 scans accumulated per sample.

The microstructure of thermally activated DT and geopolymer samples was analyzed using a scanning electron microscope (SEM, EVO 18, Carl Zeiss, Oberkochen, Germany) equipped with a scanning transmission electron microscopy (STEM) module and a PP2000T cryo-transfer system to observe the micromorphology and microstructural features of the activated tailings and geopolymer matrix. An accelerating voltage of 15 kV was applied, and all samples were gold-coated prior to imaging.

## 3. Structural Characterization of Activated Diabase Tailings

### 3.1. XRD Analysis of Activated Diabase Tailings

The mineralogical evolution of diabase tailings (DT) under stepwise thermal activation was examined to clarify phase transitions relevant to geopolymerization. Progressive decomposition of silicate-based crystalline frameworks was induced as temperature increased, revealing the thermally driven destabilization of specific mineral lattices.

**Figure 1:**
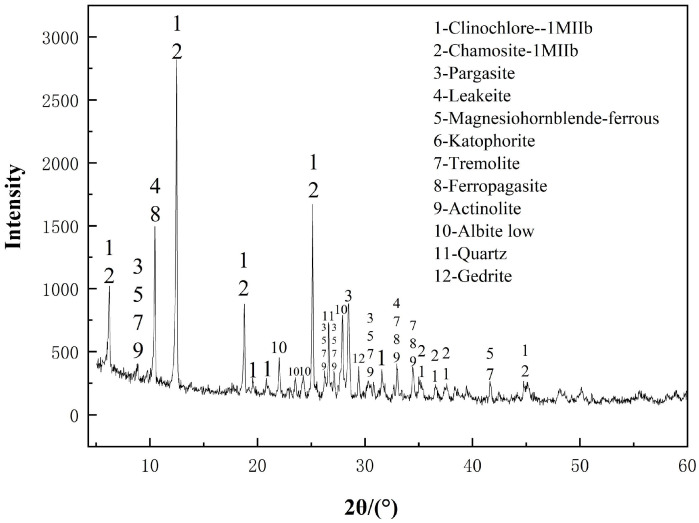
XRD Pattern of Unactivated Diabase Tailings.

X-ray diffraction patterns of raw and thermally activated DT revealed a complex assemblage of silicate minerals. The untreated material primarily comprised clinochlore (Cl), chamosite (Ch), pargasite (Par), leakeite (Lea), magnesiohornblende (Mhb), katophorite (Kat), tremolite (Tre), ferropargasite (Fpg), actinolite (Act), albite (Ab), quartz (Q), and gedrite (Ged). This mineralogical profile aligns with that reported for mafic tailings in prior studies [39]. Among these phases, layered silicates (e.g., Cl, Ch) and chain silicates (e.g., Lea, Par) demonstrated the highest thermal sensitivity. The relative intensities of amphibole- and pyroxene-related peaks declined markedly with increasing temperature, indicating progressive lattice disorder and structural breakdown. In contrast, tectosilicates such as quartz maintained their crystalline integrity even at 300 °C [40]. These phase transitions are visualized in the XRD patterns presented in Figure 1.

**Figure 2:**
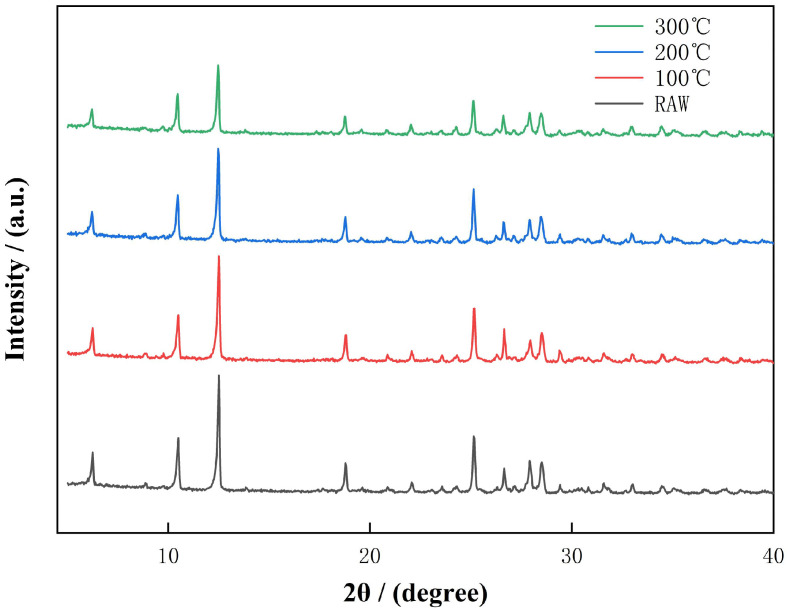
XRD patterns of diabase tailings under stepwise thermal activation conditions.

**Figure 3:**
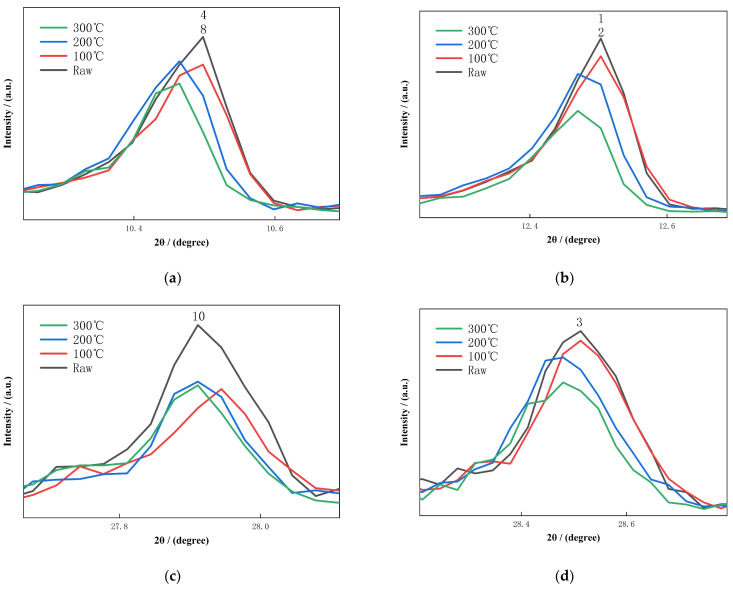
Magnified Views of Characteristic XRD Peaks during Stepwise Thermal Activation. (**a**) Magnified View of the Leakeite/Ferropargasite Peak during Stepwise Thermal Activation, (**b**) Magnified View of the Clinochlore/Chamosite Peak during Stepwise Thermal Activation, (**c**) Magnified View of the Albite Peak during Stepwise Thermal Activation, (**d**) Magnified View of the Pargasite Peak during Stepwise Thermal Activation. Numbers in the figure correspond to mineral phases as labeled in Figure 1.

To further elucidate the thermally induced transformations, selected diffraction peaks representing key minerals were magnified and analyzed. These transitions are further illustrated in the XRD patterns of diabase tailings under stepwise thermal activation, as shown in Figure 2. To more clearly illustrate the effects of stepwise thermal activation on mineral structure, the magnified views presented in Figure 3 provide a direct visualization of temperature-induced changes in intensity and diffraction peak positions of major silicate phases.

The peak corresponding to leakeite and ferropargasite, located near 10.4–10.6°, exhibited notable broadening and intensity reduction above 200 °C, as depicted in Figure 3a. This behavior suggests dehydroxylation and disruption of Fe–O and Si–O bonds within the chain silicate framework. A similar thermal response was observed for clinochlore and chamosite, whose peaks at approximately 12.4–12.6° underwent significant attenuation at 100 °C and evolved into a broad, low-intensity band by 300 °C, indicating lattice collapse and redistribution of octahedral cations, as illustrated in Figure 3b. These findings are consistent with degradation mechanisms previously reported for iron ore tailings [41].

The albite peak, centered around 27.8–28.0°, weakened progressively with increasing temperature, reflecting partial destabilization of the Na–Al–Si framework. This transformation is shown in Figure 3c. In addition, Figure 3d displays the response of pargasite, with its peak near 28.4–28.6° undergoing a similar decline in intensity due to the breakdown of the chain silicate structure. These structural deteriorations likely enhanced the availability of reactive silica and alumina species, consistent with observations in tailings-based geopolymer systems [42].

Thermal treatment at 300 °C triggered additional structural responses. Several diffraction peaks shifted toward higher 2θ angles, suggesting unit cell contraction and the formation of lattice defects. Chain silicates such as leakeite and pargasite exhibited substantial structural collapse, while tectosilicates like quartz remained largely unaffected. Tremolite showed a split peak profile at this temperature, likely resulting from Fe^3+^ redistribution within octahedral coordination sites, indicating Fe–O bond cleavage and internal lattice reorganization. These structural modifications resemble those observed in thermally activated lithium slag and quarry waste [43].

These XRD results were further corroborated by Fourier transform infrared (FTIR) spectroscopy. The attenuation of the O–H stretching band near 3400 cm^−1^ confirmed the dehydroxylation of both layered and chain silicates. Taken together, the XRD and FTIR evidence demonstrated that thermal activation induced substantial reconfiguration of the silicate framework. This restructuring increased the density of reactive surface sites and promoted the release of silico-aluminous species essential for geopolymer formation.

In summary, the thermal evolution of DT followed a sequential degradation pathway. Layered silicates such as clinochlore and chamosite underwent significant breakdown between 100 and 200 °C. Chain silicates, including leakeite and pargasite, progressively collapsed from 200 to 300 °C. Early-stage amorphization of tectosilicates such as albite occurred at 300 °C. This staged transformation—from layered to chain to framework silicates—provides a mechanistic foundation for optimizing low-temperature activation strategies to enhance the reactivity of DT in geopolymer binder systems.

### 3.2. FTIR Analysis of Activated Diabase Tailings

To further clarify the mechanisms of mineral transformation inferred from the XRD results, Fourier transform infrared (FTIR) spectroscopy was conducted to examine the evolution of hydroxyl bonding and silicate frameworks in diabase tailings (DT) subjected to stepwise thermal activation. The focus was placed on temperature-induced variations in O–H, Si–O, and Fe–O vibrational bands, which reflect structural changes in hydrous silicates, tectosilicates, and associated accessory minerals. These spectral observations were interpreted in relation to the mineral phases identified by XRD, providing complementary insights into the thermal behavior of DT.

**Figure 4:**
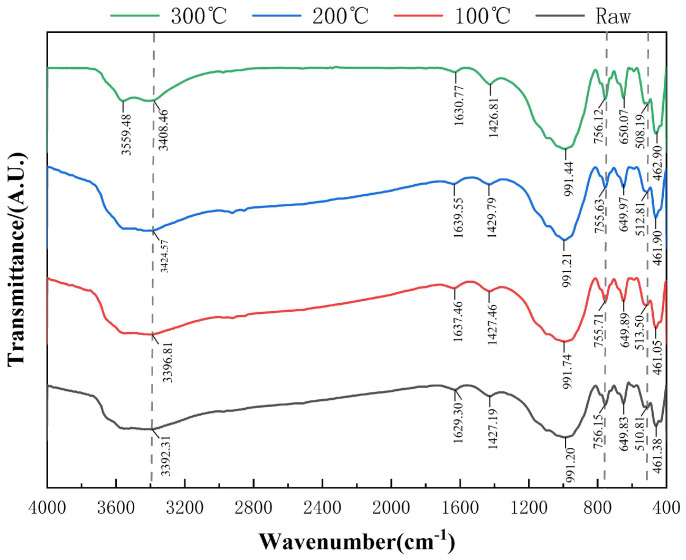
Comparative FTIR Spectra of Diabase Tailings-Based Geopolymers under Stepwise Thermal Activation.

The FTIR spectra of raw, 100 °C-, 200 °C-, and 300 °C-treated samples are presented in Figure 4. Characteristic absorption bands were assigned to mineral phases verified by XRD, including chlorite-group minerals (Cl, Ch), amphiboles (Mhb, Lea, Fpg, Ac), plagioclase (Ab), quartz (Qtz), and the carbonate mineral siderite [44].

**Figure 5:**
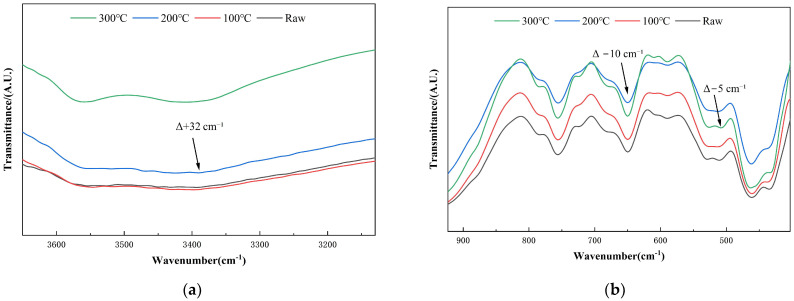
Magnified FTIR spectra of DT under stepwise thermal activation: (**a**) O–H stretching region showing a Δ + 32 cm^−1^ blueshift; (**b**) Si–O and Fe–O regions showing redshifts of Δ − 10 cm^−1^ and Δ − 5 cm^−1^, respectively.

At 100 °C, the broad O–H stretching band at 3392–3396 cm^−1^ was attributed to interlayer and adsorbed water in Cl and amphibole phases. The H–O–H bending band shifted from 1629 to 1637 cm^−1^, indicating partial loss of pore water. This redshift has been associated with the thermal desorption of physically adsorbed or interlayer water, as previously reported in FTIR studies on clay minerals [45]. A redshift in the Si–O symmetric stretching band, from 765 to 755 cm^−1^, suggested interlayer contraction within chain silicates. A minor Fe–O shift (510 → 513 cm^−1^) implied slight octahedral distortion in Mhb and magnetite. While these variations were subtle in the full-range FTIR spectrum, they are clearly resolved in the magnified views shown in Figure 5a,b.

At 200 °C, further structural changes became apparent. The O–H stretching band exhibited a distinct blueshift from 3376 cm^−1^ to 3408 cm^−1^ (Δ + 32 cm^−1^), indicating intensified dehydroxylation, as shown in Figure 5a. The Si–O band remained centered at approximately 755 cm^−1^, reflecting the structural stability of tectosilicates such as Ab and Qtz. Meanwhile, a redshift in the Fe–O vibration from 513 to 508 cm^−1^ (Δ − 5 cm^−1^), visible in Figure 5b, was attributed to partial Fe^2+^ oxidation and coordination changes. The persistence of the Fe^2+^/Mg^2+^–O vibration near 512 cm^−1^ suggested cation redistribution within amphibole octahedra. In contrast, the carbonate band (~1429 cm^−1^) exhibited negligible change (<0.5 cm^−1^), confirming the thermal resilience of siderite. These trends correspond to the gradual attenuation of amphibole reflections in the XRD patterns, indicating the progressive degradation of hydroxyl-bearing silicates [46].

By 300 °C, signs of advanced phase breakdown were evident. A new shoulder appeared in the O–H region at 3359 cm^−1^, corresponding to Fe–OH vibrations, indicating Fe/Mg coordination rearrangement, as evidenced in Figure 4. A further redshift in the Si–O band from 755 to 745 cm^−1^ (Δ − 10 cm^−1^) was observed in Figure 5b, suggesting ongoing disruption of the silicate framework. Additionally, the Si–O–Al bending vibration shifted slightly from 461 to 463 cm^−1^, indicating early framework loosening in Ab. In contrast, the antisymmetric Si–O–Si stretch (~991 cm^−1^) and carbonate bands remained nearly unchanged, suggesting preserved structural integrity in Qtz and siderite. These FTIR results are consistent with the near-complete collapse of amphibole and pyroxene peaks seen in the XRD patterns at this temperature [47].

In summary, the combined FTIR–XRD analysis revealed a sequential and phase-specific transformation mechanism. Between 200 °C and 300 °C, significant dehydroxylation and structural collapse occurred in amphiboles (Lea, Mhb, Fpg, Ac) and chlorite-group minerals (Cl, Ch). In contrast, tectosilicates (Ab, Qtz) retained their crystalline structure, while siderite exhibited excellent thermal stability. These mineralogical transformations enhanced the availability of reactive Si and Al species, thereby improving the reactivity of DT as a geopolymer precursor.

## 4. Mechanical Behavior of Diabase Tailings-Based Geopolymers (DTG)

This chapter investigates the mechanical performance of geopolymers derived from thermally activated diabase tailings, with a focus on how strength development and microstructural characteristics evolve under varying activation and curing conditions. Emphasis is placed on the synergistic effects of thermal pretreatment, curing environment, and mix design in enhancing the reactivity of the silicate-rich precursor. The correlation between mechanical strength and internal structure is systematically evaluated through compressive and flexural strength tests, supported by XRD, FTIR, and SEM analyses.

### 4.1. Analysis of Strength Development

#### 4.1.1. Effect of Thermal Activation Temperature

**Figure 6:**
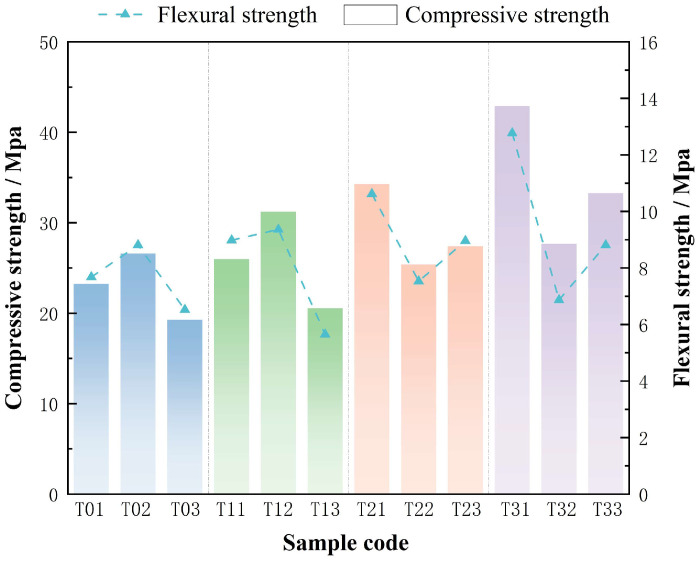
Compressive and Flexural Strengths of DTG Prepared via Stepwise Activation and Cured at Ambient Temperature.

Thermal activation is essential for enhancing the chemical reactivity of aluminosilicate precursors, particularly in low-calcium systems such as diabase tailings. In this context, the objective of this section is to clarify how controlled thermal pretreatment influences the mechanical performance of diabase tailings-based geopolymers (DTG). The enhancement is attributed to thermally induced mineral phase transitions and microstructural reorganization within the precursor matrix. Previous studies have demonstrated that moderate thermal activation can disrupt stable crystalline phases and promote the release of reactive silica and alumina species, thereby accelerating geopolymerization and improving early-age strength. However, due to the unique mineralogy of diabase tailings—dominated by amphiboles and chlorite-group minerals—the optimal activation temperature range remains uncertain. To address this, geopolymer specimens were synthesized using DT subjected to various thermal activation temperatures. After ambient curing, compressive and flexural strength tests were performed to evaluate the mechanical response of each group. A clear dependence of strength on activation temperature was observed, as illustrated in Figure 6.

In the unactivated state (T0 group), compressive strength ranged from 23.22 to 26.59 MPa, while flexural strength varied between 6.52 and 8.81 MPa. These low baseline values reflected the limited reactivity of raw DT and the incomplete formation of a geopolymeric network. Activation at 100 °C (T1 group) resulted in a marked increase in compressive strength to 31.21 MPa, representing a 17.4% improvement over the T0 group. Flexural strength also rose moderately to 9.36 MPa, yielding a flexural-to-compressive strength ratio of 0.30—exceeding the typical range of 0.25–0.28 reported for conventional slag-based geopolymers.

At 200 °C (T2 group), mechanical performance was further enhanced. Compressive strength reached 34.28 MPa, representing a 28.9% increase relative to unactivated samples, while flexural strength improved to 10.62 MPa. This corresponded to a flexural-to-compressive ratio of 0.31, indicating improved matrix toughness. As activation temperature increased, both strength indices exhibited a positive correlation with enhanced polycondensation and gel densification, attributed to the progressive decomposition of silicate minerals. Maximum performance was achieved at 300 °C (T3 group), particularly in the T31 sample, which reached a compressive strength of 42.90 MPa and flexural strength of 12.77 MPa. The resulting strength ratio (0.30) was consistent with values reported for high-performance fiber-reinforced concretes, emphasizing the synergy between optimized thermal activation and favorable chemical reactivity at moderate temperatures.

These mechanical trends were supported by XRD and FTIR analyses. XRD patterns revealed systematic mineral degradation with increasing temperature; the (001) peak of chlorite decreased by 42%, and the ferroactinolite peak disappeared entirely at 300 °C, confirming the breakdown of amphibole phases and the oxidation of Fe^2+^ to Fe^3+^. These structural transformations promoted the release of reactive Si and Al, which serve as critical precursors for geopolymer gel formation. FTIR spectra exhibited a continuous blue shift in O–H stretching vibrations (from 3392 to 3408 cm^−1^) and a red shift in Fe–O bands (from 510 to 508 cm^−1^), indicating progressive dehydroxylation and Fe oxidation. As shown in Figure 4, the emergence of an Fe–OH band at 3359 cm^−1^ and the intensification of the Si–O–Al band near 880 cm^−1^ further confirmed increased crosslinking and structural rearrangement. These changes suggest the development of Fe–O–Si and Ca–Al–Si linkages under mid- to high-temperature activation, contributing to polymer network formation. SEM analysis corroborated the chemical and mechanical findings. The unactivated matrix (T0) exhibited a porous microstructure with weak cohesion and visible microcracking. With increased activation temperature, a transition toward a denser and more homogeneous matrix was observed. Between 200 °C and 300 °C, pores became increasingly filled with amorphous gel phases. The T31 sample showed a highly compact matrix with evidence of localized fusion and thick gel coverage, consistent with the observed peak in mechanical strength.

In summary, thermal activation between 100 and 300 °C resulted in stepwise improvements in the mechanical behavior of DTG. At lower temperatures, physical dehydration dominated; at intermediate levels, Fe–O–Si bond formation was promoted; and at higher temperatures, the release of Si, Al, and Ca ions enabled the co-formation of N–A–S–H and C–A–S–H gels. Optimal performance was achieved at 300 °C with a Si/Al ratio of 2.6 under room-temperature curing. These findings demonstrate a promising pathway for the valorization of diabase tailings through low-carbon, thermally optimized geopolymer binder technologies.

#### 4.1.2. Effect of Curing Conditions

Curing conditions play a decisive role in governing the kinetics and extent of geopolymerization, particularly in low-calcium aluminosilicate systems such as those based on diabase tailings. This section aims to evaluate whether standard curing environments—defined by ambient temperature and relative humidity—can effectively enhance the mechanical performance of diabase tailings-based geopolymers (DTG). Emphasis is placed on their ability to promote phase reorganization, densify the matrix, and strengthen interfacial bonding.

**Figure 7:**
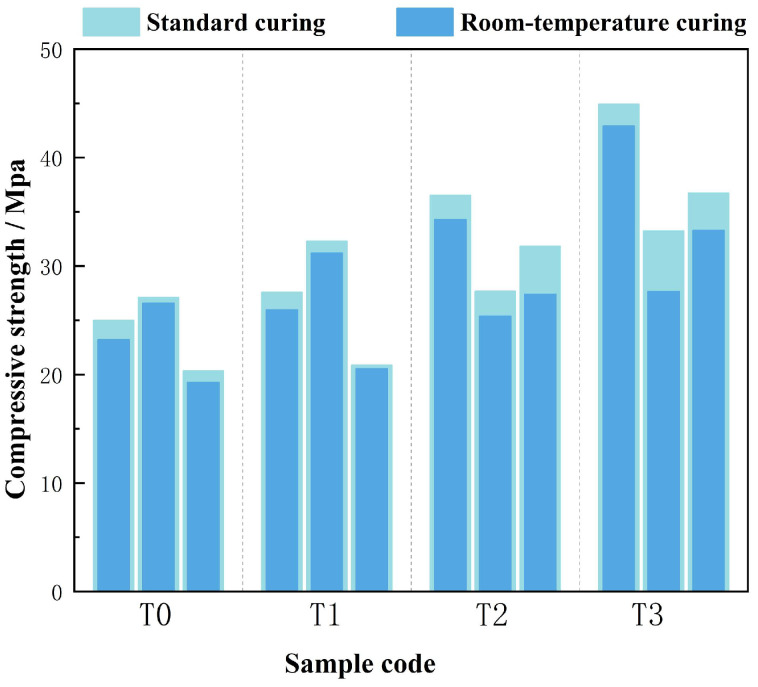
Compressive Strength of DTG under Different Curing Conditions.

**Figure 8:**
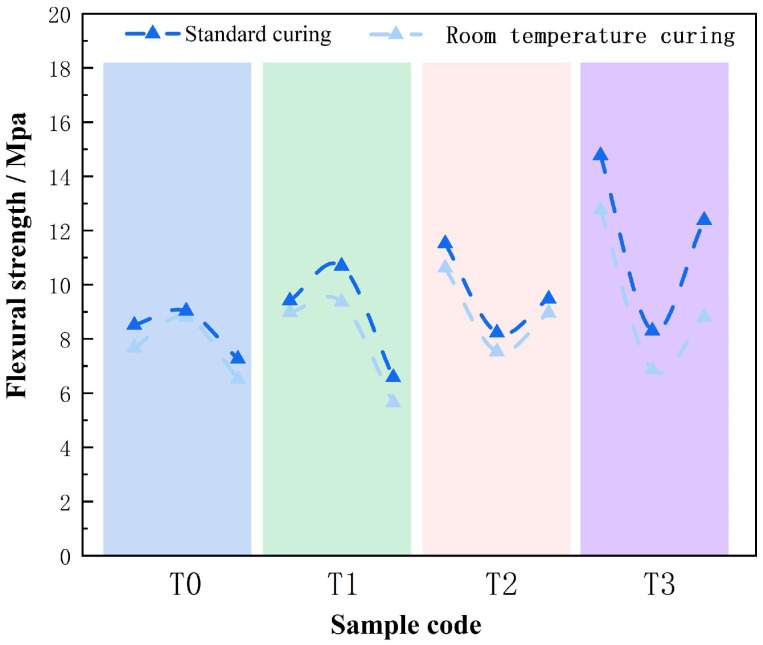
Flexural Strength of DTG under Different Curing Conditions.

Under standard curing conditions (20 ± 2 °C, 85% RH), a measurable improvement in strength was achieved. This enhancement was primarily attributed to the synergistic effects of mineral phase transitions, bond recombination, and microstructural densification. As illustrated in Figure 7, the compressive strength of the unactivated group (T0) increased by 7.78%, ranging from 24.98 to 27.11 MPa. A concurrent rise in flexural strength was recorded, with values reaching 7.26–9.03 MPa, representing a 4.85% improvement. The strength gains observed under varying curing regimes, as presented in Figure 8, aligned well with SEM observations that revealed enhanced interfacial cohesion within the matrix. Additional structural characterization by XRD and FTIR confirmed that the humid environment facilitated the hydration of residual Ca(OH)_2_ and promoted the formation of finely dispersed CaCO_3_ crystals. Nevertheless, the overall strength increase remained moderate, likely constrained by the retained crystallinity of thermally stable phases and the persistence of internal porosity.

Following thermal activation at 100 °C, more pronounced improvements in mechanical properties were observed. Flexural strength increased by 11.21%, ranging from 6.58 to 10.69 MPa, surpassing the relative gain in compressive strength. FTIR spectra showed stabilization of the Fe–O band at 510 cm^−1^ and the emergence of an Fe–OH vibration at 3359 cm^−1^, indicating increased dispersion of Fe^3+^ species. SEM analysis revealed that CaCO_3_ crystals filled interfacial voids and microcracks, enhancing cohesion between gel phases and residual particles. As a result, the flexural-to-compressive strength ratio increased to 0.33, exceeding the typical range for conventional geopolymers and indicating improved ductility and matrix integrity.

At 200 °C, the effects of standard curing became increasingly dependent on activation temperature. Compressive strength rose by 7.82–10.03%, reaching 27.69–36.52 MPa, while flexural strength increased by 8.23–11.52%. XRD patterns revealed further disruption of chlorite interlayers, and FTIR shifts at 991 and 461 cm^−1^ suggested intensified reactivity of silica and alumina species. SEM images showed progressive pore-filling by newly formed gels, reducing porosity from 35% to 30%. The BT21 sample exhibited a flexural strength of 11.52 MPa, with a flexural-to-compressive strength ratio of 0.32 (Figure 7), consistent with the development of Fe–O–Si networks and improved matrix cohesion under controlled humidity.

Thermal activation at 300 °C resulted in the most significant mechanical enhancement. Compressive strength increased by 10.65–24.60%, while flexural strength improved by 16.65–24.60%. XRD analysis showed a 42% reduction in the chlorite (001) peak and complete disappearance of ferroactinolite, confirming substantial Fe^2+^ oxidation to Fe^3+^. FTIR spectra indicated stabilization of the 508 cm^−1^ band, reflecting strengthened interfacial bonding between Fe_2_O_3_ and the surrounding gel. SEM revealed a 50% reduction in pore volume, and the formation of dense C–A–S–H gels increased CaO utilization from 75% to 92%. The BT31 sample achieved the highest mechanical performance, with a compressive strength of 44.93 MPa and a flexural strength of 14.77 MPa. The resulting flexural-to-compressive strength ratio of 0.33 was comparable to that of engineered cementitious composites (Figure 6 and Figure 7). Even in Al^3+^-deficient systems such as T32, standard curing led to a 20.8% increase in flexural strength, from 6.87 to 8.30 MPa. This improvement was supported by FTIR spectra showing broadening of the Fe–O vibration band at 508 cm^−1^ without peak splitting, suggesting partial recovery of network distortion and improved continuity within the Fe–O–Si framework.

In summary, standard curing significantly enhanced DTG performance by facilitating mineral decomposition, promoting recombination of Fe, Si, Al, and Ca bonds, and improving gel distribution throughout the pore network. The combination of thermal activation and humidity control raised the flexural-to-compressive strength ratio by 8.5% at moderate temperatures and increased CaO utilization to 92% at 300 °C. These results provide mechanistic insight and practical guidance for optimizing low-carbon geopolymer binder systems based on diabase tailings.

#### 4.1.3. Statistical Analysis of Orthogonal Design

To further clarify the strength evolution under multifactor interactions, a statistical evaluation based on an orthogonal experimental design was performed. The individual contributions of activation temperature, Si/Al molar ratio, and curing condition to the compressive strength of DTG were quantified through mean response analysis, main effect plots, and analysis of variance (ANOVA).

**Figure 9:**
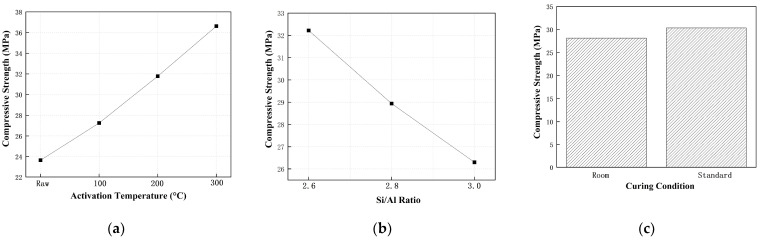
Main effect plots showing the impact of (**a**) activation temperature, (**b**) Si/Al molar ratio, and (**c**) curing condition on the compressive strength of diabase tailings-based geopolymers (DTG).

The average compressive strength increased markedly with rising activation temperature, from 26.58 MPa in the unactivated sample to 36.62 MPa at 300 °C, as illustrated in Figure 9a. This enhancement corresponds to the progressive breakdown of silicate minerals—particularly chlorite and amphibole—and the increased availability of reactive silica and alumina, which favor the formation of N–A–S–H and C–A–S–H gel phases.

As shown in Figure 9b, the effect of the Si/Al ratio followed a nonlinear trend. Maximum compressive strength was achieved in the range of 2.6–2.8, beyond which a slight decline was observed. This reduction may result from an imbalance in the alkali activation environment at higher silica contents, potentially hindering optimal gel formation.

Standard curing (20 °C, RH 95%) led to slightly higher strength than ambient conditions, likely due to enhanced hydration and gel polymerization under elevated humidity, as demonstrated in Figure 9c.

These trends are further reinforced in the overall main effect plots in Figure 9. Among the three factors, activation temperature exhibits the steepest slope, confirming its dominant influence. The slope associated with the Si/Al ratio is moderate, while that for the curing condition remains comparatively flat, suggesting a weaker effect.

The ANOVA results presented in Table 3 statistically substantiate these findings. Activation temperature was identified as the most significant factor, with a sum of squares (SS) of 562.276, an F-value of 10.737, and a *p*-value of 0.0003 (*p* < 0.001). The Si/Al ratio also showed a statistically significant effect (SS = 158.325; F = 4.535; *p* = 0.0264). In contrast, the effect of the curing condition was not significant (SS = 29.040; F = 1.664; *p* = 0.2144), reflecting its minor contribution.

The residual variation (SS = 296.764; df = 17) accounts for unexplained fluctuations, likely originating from random error or minor interaction effects not captured in the orthogonal matrix. Overall, these results confirm that thermal activation plays a decisive role in the decomposition of inert silicate phases and the generation of reactive species, thereby governing the strength development of DTG.

### 4.2. Microstructural Characterization by SEM

#### 4.2.1. Microstructural Features at Different Activation Temperatures

**Figure 10:**
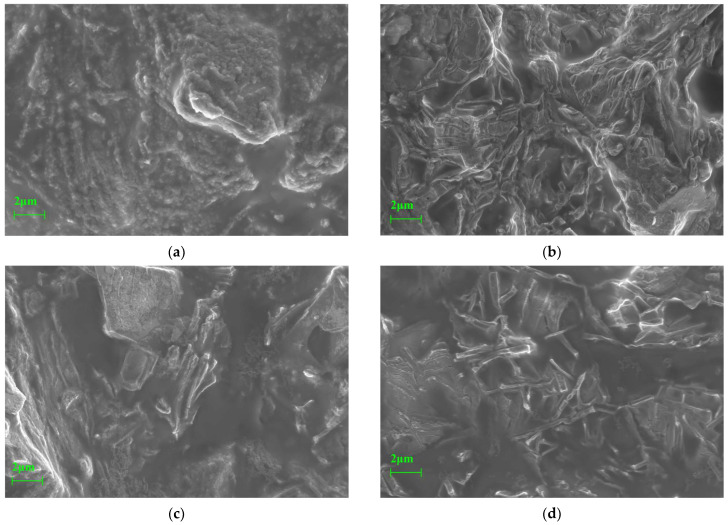
Microstructural analysis of DTG prepared from stepwise-activated DT: (**a**) Group T01; (**b**) Group T11; (**c**) Group T21; (**d**) Group T31.

Thermal activation has been widely employed to modify the microstructure of aluminosilicate precursors and promote the generation of reactive phases in geopolymer systems. In the present study, the microstructural evolution of diabase tailings-based geopolymers (DTG) was examined under varying activation temperatures to determine how thermal pretreatment influences phase morphology, particle contact, and interfacial bonding. Emphasis was placed on the relationship between microstructural characteristics and mechanical performance, supported by phase analyses confirmed by XRD and FTIR. These observations are presented in Figure 10, which illustrates the microstructural changes in DTG prepared from stepwise-activated diabase tailings.

As illustrated in Figure 10a, the DTG matrix in the unactivated state (T01 group) exhibited a coarse, angular, and highly irregular morphology. Mineral particles featured rough surfaces with abundant open pores and limited interparticle adhesion. No reaction gel was observed, and distinct particle boundaries remained clearly defined, indicating insufficient geopolymerization. XRD patterns confirmed the dominance of residual crystalline phases, marked by sharp peaks corresponding to clinochlore (2θ ≈ 16.2°), pyroxene (2θ ≈ 28.5°), and quartz (2θ ≈ 26.6°). FTIR spectra revealed strong Si–O–Si asymmetric stretching near 991 cm^−1^ and Fe–O vibrational bands around 589 cm^−1^, while broad O–H absorption persisted, suggesting minimal dehydroxylation and limited structural reorganization. As a result, mechanical strength in the unactivated sample was largely attributed to physical packing rather than chemical bonding. The compressive and flexural strengths ranged from 23.22 to 26.59 MPa and 6.52 to 8.81 MPa, respectively.

Following thermal activation at 100 °C, early-stage reactivity became evident in the T11 group, as shown in Figure 10b. Flocculent and reticulated gel phases partially enveloped residual mineral particles. However, large angular pores persisted, indicating incomplete gel precipitation and insufficient matrix densification. XRD patterns revealed reduced intensities of clinochlore and pyroxene peaks, along with the emergence of a broad amorphous hump between 2θ = 20–35°, suggesting partial structural degradation. FTIR spectra showed broadening of the –OH band at ~3408 cm^−1^ and a transition in Al–O coordination from octahedral to tetrahedral environments, reflecting enhanced dissolution activity. Despite these changes, the strong Si–O–Si band at 991 cm^−1^ remained, indicating that the chlorite framework was only partially disrupted. As a result, flexural strength increased moderately to 9.36 MPa, with the flexural-to-compressive strength ratio remaining at 0.30, highlighting limited matrix cohesion.

At 200 °C, substantial microstructural refinement was observed in the T21 group, as illustrated in Figure 10c. The matrix evolved into a compact framework composed of interlocked blocky and acicular crystals embedded within fibrous and lamellar gels. Previously open pores were partially filled with new gel phases, enhancing continuity and mechanical interlocking. XRD analysis showed the disappearance of clinochlore and chamosite reflections, replaced by a dominant amorphous background, indicating extensive depolymerization. FTIR spectra revealed attenuation of the carbonate band at 1427 cm^−1^ and suppression of the Si–O–Si vibration. A new peak at ~880 cm^−1^ associated with Si–O–Al bonding emerged, while a shift in the Fe–O band near 510 cm^−1^ indicated reconfiguration of Fe^3+^ coordination and formation of Fe–O–Si linkages. These changes corresponded with a compressive strength increase to 34.28 MPa—representing a 28.9% improvement over the unactivated sample. However, a further increase in the Si/Al ratio to 3.0, as in the T23 group, led to reduced performance, underscoring the need to maintain an optimal ratio near 2.6 for structural stability.

Thermal activation at 300 °C resulted in a well-developed and homogeneous matrix in the T31 group, as depicted in Figure 10d. The microstructure was dominated by lamellar and fibrous gel networks, with minimal visible porosity. Residual crystalline features were nearly absent, suggesting complete depolymerization and structural reorganization of the silico-aluminous phases. XRD patterns exhibited the disappearance of all sharp diffraction peaks, replaced by a broad amorphous halo, confirming full phase transformation. FTIR analysis showed a downshift of the –OH band from 3392 to 3359 cm^−1^, intensification of the Si–O–Al vibration at 880 cm^−1^, and near elimination of the Si–O–Si band at 991 cm^−1^. A stronger Si–O bending mode at 461 cm^−1^ reflected increased structural ordering within the gel. These spectral changes confirmed the coexistence of N–A–S–H and C–A–S–H gels, which contributed to the formation of a dense, interconnected matrix. The T31 sample achieved compressive and flexural strengths of 42.9 MPa and 12.77 MPa, respectively. When the Si/Al ratio was adjusted to approximately 2.8, shrinkage-induced microcracking was mitigated, further improving mechanical performance [48].

Overall, the results demonstrated that stepwise thermal activation facilitated the depolymerization of mineral phases, enhanced the dissolution of reactive species, and promoted the formation of a cohesive gel matrix. The progressive microstructural evolution observed across Figure 10a–d was closely aligned with improvements in mechanical strength, confirming the effectiveness of low-temperature activation in transforming diabase tailings into high-performance geopolymer binders.

#### 4.2.2. Microstructural Features Under Different Curing Conditions

**Figure 11:**
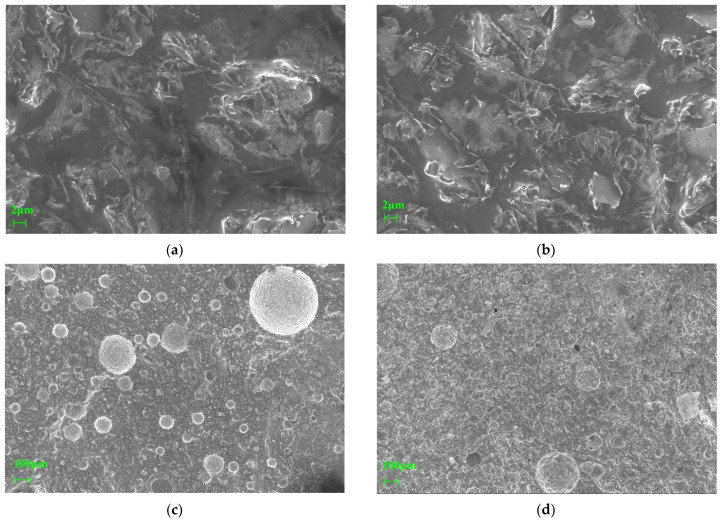
SEM images of DTG under different curing conditions: (**a**) Group T31 at 2k×; (**b**) Group BT31 at 2k×; (**c**) Group T31 at 50×; (**d**) Group BT31 at 50×.

Curing conditions have a pronounced impact on pore development and gel continuity in low-calcium geopolymer systems. To evaluate these effects, the microstructure of DTG samples subjected to ambient and sealed curing was examined using scanning electron microscopy (SEM). Figure 11 presents a comparative set of SEM micrographs, highlighting morphological differences in phase distribution, porosity, and interfacial cohesion under the two curing regimes.

Under ambient conditions, the T31 group displayed a heterogeneous dispersion of columnar and rod-like crystalline phases, as illustrated in Figure 11a. These crystalline domains were loosely embedded in the surrounding gel matrix, and poorly defined interfaces were observed, suggesting inadequate interfacial bonding. This discontinuous microstructure was attributed to kinetic instability induced by fluctuations in ambient humidity, which disrupted the sustained development of a coherent gel network. At lower magnification, Figure 11c reveals a dense array of interconnected macropores, occupying nearly 38% of the matrix volume. This high porosity significantly compromised structural integrity and impeded efficient stress transfer across the material.

In contrast, the BT31 group, which underwent standard curing under sealed, high-humidity conditions, displayed markedly improved microstructural features, as shown in Figure 11b. The density of columnar crystals increased by approximately 40% and was more uniformly distributed throughout the matrix. Interfacial bonding between the gel phase and embedded crystals appeared substantially enhanced, suggesting stronger chemical integration. As shown in Figure 11d, this improved bonding was accompanied by a significant reduction in both average pore size and total porosity. Mercury intrusion porosimetry confirmed a decrease in macropore volume, a narrower pore size distribution, and a marked reduction in closed micropores. These refinements were attributed to the stabilization of ionic dissolution and polycondensation reactions under constant humidity, which minimized shrinkage-induced cracking and promoted the uniform development of the gel phase. These microstructural improvements translated directly into enhanced mechanical performance. The BT31 sample achieved a compressive strength of 44.93 MPa, representing a 4.7% increase over the T31 group. Flexural strength increased more substantially—by 15.7%—reaching 14.77 MPa, while the flexural-to-compressive strength ratio rose from 0.30 to 0.33. This shift indicated improved ductility and energy dissipation capacity, driven by the denser matrix and enhanced cohesion within the gel phase.

FTIR analysis provided additional support for these findings. The Si–O–Al vibration band at 880 cm^−1^ increased in intensity by 19%, reflecting the formation of a more extensive and chemically integrated N–A–S–H gel framework. Simultaneously, the full width at half maximum of the Fe–O band near 510 cm^−1^ decreased by 32%, indicating greater amorphization and the establishment of Fe–O–Si linkages. These spectral changes corroborated the observed densification of the matrix and strengthened interfacial bonding. Curing conditions also exerted a pronounced influence on calcium phase behavior. XRF analysis confirmed that the 10.05 wt% CaO present in the BT31 group was fully hydrated under standard curing, resulting in the formation of C–A–S–H gel. This transformation was supported by a 17% increase in the FTIR signal near 1100 cm^−1^. Under room-temperature curing, however, a portion of the CaO was converted into Ca(OH)_2_, which subsequently underwent carbonation, as evidenced by an 8% increase in the CO_3_^2−^ band at 1427 cm^−1^. This carbonation process contributed to interfacial microcracking and a reduction in overall bonding strength.

In summary, standard curing reduced the total porosity of the DTG matrix to below 9% and enabled compressive strengths exceeding 44 MPa, in accordance with the cement performance requirements specified in the Chinese National Standard GB 175-2023 I 42.5R [49]. Notably, the relative improvement in flexural strength outpaced that of compressive strength, underscoring the pivotal role of humidity control in mitigating brittleness and enhancing the ductility of diabase tailings-based geopolymer matrices [50].

## 5. Conclusions

The formation mechanism and performance enhancement of diabase tailings-based geopolymer (DTG) were systematically clarified under the dual influence of low-temperature thermal activation (100–300 °C) and humidity-controlled curing. A sequential mineral transformation—consisting of interlayer dehydration, hydrogen bond disruption, and dehydroxylation—was found to progressively degrade the crystalline frameworks of chlorite and amphibole. This degradation promoted the release of reactive Si, Al, and Ca species, which subsequently participated in the formation of a highly cross-linked N–A–S–H and C–A–S–H gel matrix, contributing to improved chemical continuity and microstructural integrity.

The optimal mechanical performance was achieved at an activation temperature of 300 °C and a Si/Al molar ratio of 2.6. Under these conditions, the DTG exhibited a compressive strength of 42.9 MPa and a flexural strength of 12.77 MPa. Standard curing (20 ± 2 °C, 95% RH) further enhanced matrix densification through the formation of Fe–O–Si bonds and co-precipitation of aluminosilicate gel phases. These structural improvements raised the flexural-to-compressive strength ratio to 0.33, reflecting enhanced fracture resistance and mechanical toughness beyond that of conventional cement-based materials.

This dual-regulation strategy provides a practical route for converting low-reactivity diabase tailings into high-performance, low-carbon binders. The combined thermal-curing pathway defines a direct process–structure–property correlation, whereby thermal decomposition governs gel chemistry and humidity regulation improves pore morphology and interfacial cohesion. These findings establish both mechanistic insight and engineering guidance for the sustainable reuse of silicate-rich tailings in next-generation construction materials and scalable green infrastructure applications.

Future investigations should prioritize the long-term durability assessment of DTG under diverse service conditions, with particular attention to strength development over curing age and water stability under wet–dry cycling or immersion exposure. To gain deeper insight into gel structure and pore evolution, advanced characterization techniques—such as nuclear magnetic resonance (NMR), synchrotron-based X-ray absorption spectroscopy, and micro-computed tomography (micro-CT)—are strongly recommended. In addition, the synergistic effects of supplementary additives and fiber reinforcement should be explored to further tailor DTG performance for specific engineering applications.

## Figures and Tables

**Table 1:** Chemical Composition of Diabase Tailings (wt%, normalized to dry basis, LOI excluded).

Chemical Composition	SiO_2_	Fe_2_O_3_	Al_2_O_3_	CaO	MgO	TiO_2_	Na_2_O	K_2_O	MnO
Tailings/(wt%)	49.05	16.99	15.6	10.05	5.05	0.91	0.78	0.73	0.36
Chemical Composition	P_2_O_5_	SO_3_	CuO	PdO	Rh	SrO	ZrO_2_	Rb_2_O	/
Tailings/(wt%)	0.14	0.14	0.05	0.05	0.04	0.02	0.01	0.01	/

**Table 2:** Orthogonal Experimental Design for DTG Formulations.

NO.	Activation Temperature (°C)	Silicate Modulus	Si/Al	Curing Condition
T01	Raw	1.2	2.6	Room-temperature curing
T02	Raw	1.2	2.8	Room-temperature curing
T03	Raw	1.2	3.0	Room-temperature curing
BT01	Raw	1.2	2.6	Standard curing
BT02	Raw	1.2	2.8	Standard curing
BT03	Raw	1.2	3.0	Standard curing
T11	100	1.2	2.6	Room-temperature curing
T12	100	1.2	2.6	Room-temperature curing
T13	100	1.2	2.8	Room-temperature curing
BT11	100	1.2	3.0	Standard curing
BT12	100	1.2	2.6	Standard curing
BT13	100	1.2	2.8	Standard curing
T21	200	1.2	3.0	Room-temperature curing
T22	200	1.2	2.6	Room-temperature curing
T23	200	1.2	2.8	Room-temperature curing
BT21	200	1.2	3.0	Standard curing
BT22	200	1.2	2.6	Standard curing
BT23	200	1.2	2.8	Standard curing
T31	300	1.2	3.0	Room-temperature curing
T32	300	1.2	2.6	Room-temperature curing
T33	300	1.2	2.8	Room-temperature curing
BT31	300	1.2	3.0	Standard curing
BT32	300	1.2	2.6	Standard curing
BT33	300	1.2	2.8	Standard curing

**Table 3:** Average response values and ANOVA results for the effects of activation temperature, Si/Al ratio, and curing condition on the compressive strength of DTG.

Factor	Sum of Squares (SS)	Degrees of Freedom (df)	F-Value	*p*-Value
Activation Temp	562.276	3	10.737	0.0003
Si/Al Ratio	158.325	2	4.535	0.0264
Curing	29.04	1	1.664	0.2144
Residual	296.764	17	/	

## Data Availability

The original contributions presented in this study are included in the article. Further inquiries can be directed to the corresponding authors.
